# Searching for Carlos Torre Repetto: the enigmatic life of a Mexican chess prodigy

**DOI:** 10.1590/0004-282X-ANP-2021-0010

**Published:** 2021-05-08

**Authors:** Amado JIMÉNEZ-RUIZ, Adriana RUIZ-RAZURA

**Affiliations:** 1 Western University, Department of Neurology, London, Ontario, Canada. Western University Department of Neurology London Ontario Canada; 2 Universidad de Guadalajara, Centro Universitario de Arte, Arquitectura y Diseño, Guadalajara, Jalisco, México. Universidad de Guadalajara Universidad de Guadalajara Centro Universitario de Arte, Arquitectura y Diseño Guadalajara Jalisco Mexico

We thoroughly enjoyed the article by Leite Franklin et al. titled *Neurology, psychiatry*, *and the chess game: a narrative review*^1^.

In his book, *Homo Ludens*, the Dutch historian Johan Huizinga considered the element of play to be as essential as reflection for all human beings. According to Huizinga, games enhance various qualities: they create order by having rules and limits in time and space, foster social relationships, simulate reality, and captivate by generating tension and competitiveness^2^. Undoubtedly, all these characteristics are present in chess, but any activity taken to the extreme might trigger mental dysfunction when done excessively. As chess *aficionados*, we would like to enrich the conversation by sharing insight into a forgotten figure from our national memoirs, who has an enigmatic history of psychiatric disease.

Carlos Torre Repetto (1904-1978) was the greatest chess player in Mexican history (Figure 1). He started playing chess at five years old in his hometown in Merida (Yucatan, Mexico). His family fled the country a few years later to avoid the Mexican Revolution of 1910. He spent his formative years improving his game in New Orleans and other chess clubs in the United States.

**Figure 1.**
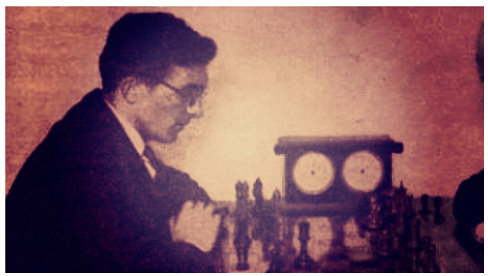
Mexican chess grandmaster, Carlos Torre Repetto.

At a young age, he participated in the acclaimed Moscow tournament, in which he tied with champion José Raúl Capablanca and defeated Emmanuel Lasker. He would rise to become one of the best players of his time, traveling worldwide and eventually becoming the first Mexican chess player to be awarded the title of International Grandmaster by the World Chess Federation in 1977^3^.

Torre Repetto joins the ranks of a long list of chess players who suffered from mental health issues. At the young age of 22, he retired from tournaments due to a “nervous breakdown” probably triggered by stress, burnout, romantic deception, and economic turmoil^4^.

Impoverished, Torre Repetto spent the last years of his life in a nursing home in Merida. In an interview conducted in 1975, a journalist asked Torre Repetto why he retired from chess with such a promising future ahead, to which he answered:

“*Well, my brother asked me to help him out in his drugstore and told me I could make some free money. I never spent a cent, but the work in the drugstore challenges the brain less than chess does. Also, I retired from competition, not from study. To this day, I still study the game*”^5^*.*

The chess opening known as the *Torre Attack* is named in his honor. The *Torre Memorial,* an annual chess tournament played in Merida since 1987, is still played today.

When asked if chess was science or an art, Torre Repetto responded:

“*Chess is science because it has its own standards, its precise mathematical mechanism whose errors are quite tangible and in which the best paths are progressively discovered, and the wrong variants are technically checked. But it is also art; there is not a single path - or a best one - to follow, but each path fits the personality of its author, and therefore, it is a way of expressing beauty, for which passion and true inspiration are required.*”

## References

[1] 1. Franklin GL, Pereira BNGV, Lima NSC, Germiniani FMB, Camargo CHF, Caramelli P, et al. Neurology, psychiatry and the chess game: a narrative review. Arq Neuro-Psiquiatr. 2020 Mar;78(3):169-75. https://doi.org/10.1590/0004-282x20190187. 10.1590/0004-282x20190187 32348415

[2] 2. Huizinga, Johan. Homo Ludens. Switzerland: Routledge; 1944.

[3] 3. Raúl OV, Medellín Anaya A. Carlos Torre Repetto “Genio, Legado y leyenda.” Ciudad de México: Editorial Apolo; 2015.

[4] 4. Velasco G. The Life and Chess Games of Carlos Torre: Mexico’s First Grandmaster. Milford, CT: Russell Enterprises, Inc.; 2016.

[5] 5. Cámara Patrón A. Carlos Torre Repetto: un grande del ajedrez. Rev Univ Autónoma Yucatán. 2011 Ene/Jun;256(1):3-9.

